# Chinese expert consensus on clinical prevention and treatment of scar^+^

**DOI:** 10.1186/s41038-018-0129-9

**Published:** 2018-09-17

**Authors:** Kaiyang Lv, Zhaofan Xia, On behalf of the Chinese consensus panel on the prevention and treatment of scars

**Affiliations:** Department of Burns, Changhai Hospital affiliated to Navy Medical University, No. 168 Changhai Road, Yangpu District, Shanghai, 200433 China

**Keywords:** Hypertrophic scar, Keloids, Scar assessment, Scar prevention, Scar treatment, Laser, Burn, Consensus, Trauma

## Abstract

Following injury, Asian skin has a tendency toward hyperpigmentation and scar formation than Caucasians. A standardized algorithm tailored to Asian patients, especially Chinese patients, is in great demand. Twelve independent, self-selected academic and military physicians from the department of burn/trauma, plastic surgery and dermatology with extensive experience in treating scars were assembled on January 17, 2015, establishing the consensus panel. This consensus was then appraised, drafted, reviewed, and finalized during the following 3 years, aiming to standardize and improve scar prevention and treatment in China. Hopefully, it may also provide some advices and references for the management of scarring in Asian patients.

## Background

Characterized by morphological and histopathological changes of normal skin following varied types of injuries, scar is considered to be an essential component of the wound healing process [1, 2]. An imbalance between destruction and deposition of collagen induced by various factors during wound healing leads to the formation of pathological scar [3, 4]. Scarring that manifested changes to the appearance and function impairs patients’ life, both physically and psychologically, and in some severe cases, inflicting patients’ self-confidence, making them feel inferior [3, 5]. Therefore, scar is becoming a clinical focus in the department of burn/trauma, plastic surgery, and dermatology.

Objective and reliable methods on scar assessments as well as strategies on scar prevention and treatment are focused clinical areas. Most of the treatments have been proved to be effective during the past two decades, while few were supported by prospective studies designed with control groups. In some cases, even safety data are lacking. Several novel therapies showed early-phase efficacy in trials with a small sample size, yet these results have not been proved in larger trials with long-term follow-up [6, 7]. In recent years, the understanding of wound healing and scar formation is being deepened, the clinical experience in scar treatment is being accumulated, and novel agents and new therapeutic options are being developed. Moreover, some traditional treatment concepts are overturned by emerging technologies, thus making it necessary to develop a standardized algorithm that is safe and effective to guide the clinical practice.

In 2002, an international advisory panel, consisting of experts from America, Italy, Germany, etc., first published “International clinical recommendations on scar management” in *Plastic and Reconstructive Surgery* [8]. In 2014, the original advisory panel published “Updated international clinical recommendations on scar management” in *Dermatologic Surgery* [9, 10]. By assessing new clinical evidence, advisory panel members have affirmed the emerging treatment options with substantial supporting data such as bleomycin, onion extract, mitomycin, and imiquimod. Considerable studies have found that a darker skin increases the risk of scar formation [11, 12]. Asians are more susceptible to hypertrophic scars than Caucasians [13]. This suggests great differences in pathogenesis of scarring between Asians and Caucasians. Asian skin is characterized by excessive fibroblast participation and collagen deposition during wound healing [11]. As a result, there is a tendency toward scar formation and hyperpigmentation after skin injury, and it takes longer time to mature (prolonged hyperemia during scar maturation) [11, 12]. Therefore, the abovementioned “International clinical recommendations on scar management” written for Caucasians is not entirely suitable to Asians.

Taking “Updated international clinical recommendations on scar management” as reference, this consensus is based on clinical evidence and adjusted to China’s national conditions and clinical practice. After an extensive discussion, the Chinese consensus panel has developed recommendations on scar prevention and treatment, which is applicable in China, expecting to standardize and improve the scar management. The mandarin version of consensus has been published on *Chinese Journal of Injury Repair and Wound Healing*, December 2017, volume 12, No 6 [14].

## The understanding of scar formation

Scar is characterized by morphological and histopathological changes of normal skin caused by various skin injuries. Moderate scarring is a normal manifestation of wound repair, which is an important part of human’s self-defense, while excessive scarring should be regarded as morbidity [15].

Though the pathogenesis of scar has not been fully understood, relevant cognitive exploration has been deepened on both micro and macro level. On the micro level, it not only involves the interaction between cells (fibroblasts, myofibroblasts, mast cells, neutrophils, etc.), cytokines (transforming growth factor β, tumor necrosis factor α, endothelial growth factor), extracellular matrix (collagen metabolism and disordered arrangement, changes in glycosaminoglycans), and other components, but also may involve the three-dimensional organization of the spatial histological structure (repair of the spatial regulatory networks formed among cells, etc.) in the whole process of scar formation [16–18]. On the macro level, diverse factors, including individual demographic characteristics (race, gender, age, etc.) and external factors (injury, surgical incisions and other treatment factors, etc.), have great influences on the scar formation [19]. Complex factors arising from multi aspects make the complexity and diversity of scar formation. Consequently, an in-depth understanding of the scar pathogenesis should benefit health care providers to achieve more accurate scar classification and optimized strategy for scar management, thus establishing a basis for more effective clinical prevention and treatment of scars.

## Scar classification

Currently, there is no standard approach for scar classification in clinical practice.

Based on color, texture, and patients’ feeling, scars can be classified into two types, immature and mature. Immature scar usually occurs in early phase of wound healing process, characterized by focally red, visible extensive capillaries on the surface, thick from several millimeters to several centimeters, rough surface, hard texture, and poor flexibility. Sometimes, it may be itchy or painful or accompanied by other obvious discomforts. Scar takes time to mature, generally about 1 year, in some cases a few years. The mature scar or scar maturation is featured by lighted-color similar to the surrounding skin, no extensive capillaries on the surface, thin thickness, soft texture, and absence of uncomfortable symptoms [10].

Based on anatomy, scars can be classified as hypertrophic scars, keloids, atrophic scars, and scar carcinomas. Hypertrophic scar is the most common type in clinical practice and can be subdivided by clinical features. Linear hypertrophic scar (e.g., surgical or traumatic) and widespread hypertrophic scar (e.g., burn or traumatic) are common subtypes in clinical scenarios [3, 10]. Keloid, in contrast, is a special type of pathological scar. Keloids grow above the surface of normal skin and beyond the edge of the initial wound and appear as continuous growing mass with hard texture that is less flexible, itchy or painful, manifesting tumor-like features as refractory to treatment and a high probability of recurrence. Keloids can be roughly divided into “inflammatory type” and “tumorous type” according to its pathogenesis. The former usually presents with symptoms like higher blood perfusion and itch, while the latter appears as insignificant blood perfusion, dark color, and apparent lump which is similar to tumor. Atrophic scars are recessed below the skin surface, exhibiting generalized cutaneous atrophy resulting from loss or contracture of cutaneous collagen fibers, usually occurring after acne infections and trauma [20]. Scar carcinomas are aggressive and malignant tumors that occur in scarring skin, also known as Marjolin’s ulcer. Burn injury is its most common etiology in clinical practice [21].

There are some major differences between hypertrophic scar and keloid. A hypertrophic scar is firm, raised within the site of injury, occasionally symptomatic, and usually develops within 4 to 8 weeks of injury. These scars typically form over extensor joints and other areas of high tension. Although it may take years, hypertrophic scars tend to regress over time (become flatter and more pliable). Unlike hypertrophic scars, keloid scars can appear many years later and extend beyond the site of injury. Keloid scars are raised reticular dermal lesions that spread beyond the confines of the original wound and invade the surrounding healthy skin. They can develop up to, or even beyond, 1 year after the injury and do not tend to regress spontaneously [22, 23].

## Scar assessment

Validated scar assessment can guide the clinical treatment, track the development and outcome of scars, and relieve patient’s concerns on the prognosis of scars. Currently, the most commonly used scales are as follows.

**Fig. 1:**
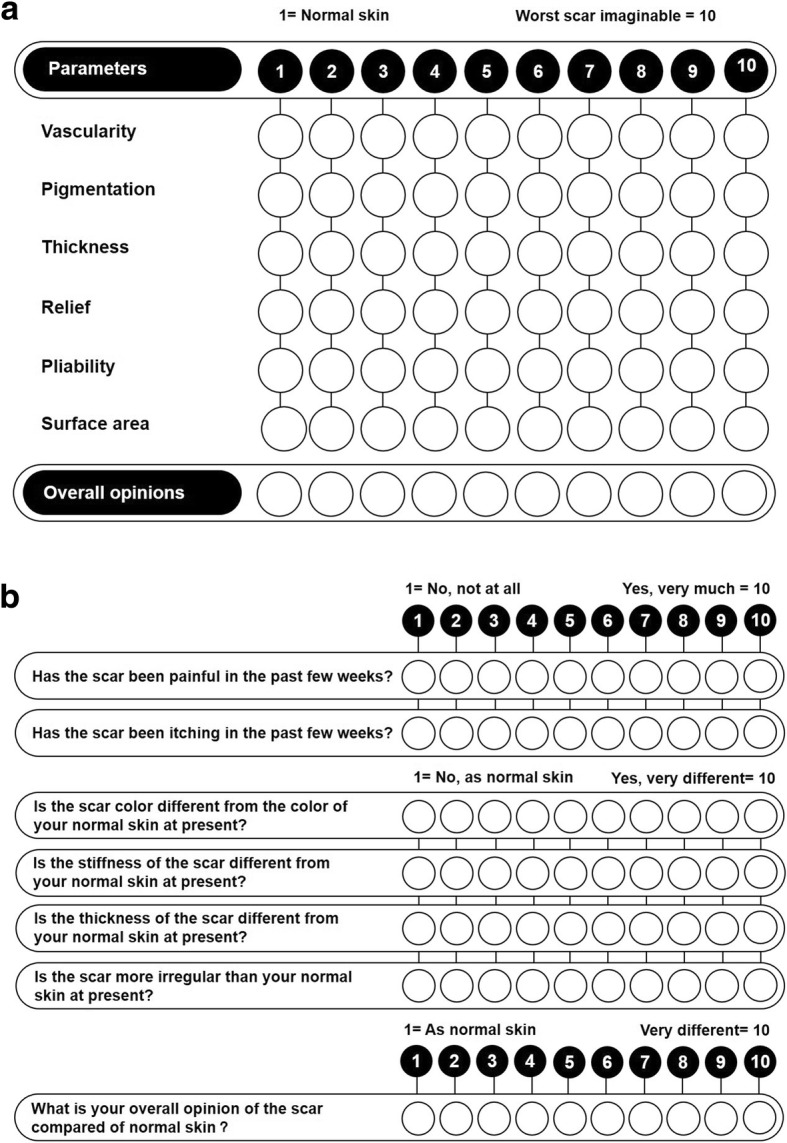
The Patient and Observer Scar Assessment Scale. **a** The Observer Scar Assessment Scale. **b** The Patient Scar Assessment Scale

1. Vancouver Scar Scale (VSS) [24]
  VSS, a widely used scale around the world, can be applied through observer’s visual observation and hand palpation instead of special equipment. The assessment covers four dimensions: pigmentation, thickness, vascularity, and pliability. VSS is characterized by user convenience and comprehensive variables, thus being wildly applied for assessment on hypertrophic scars after burn (Table 1).
2. Visual Analog Scale (VAS) [25]
  VAS is a photograph-based scoring system. Scar can be rated in this scale in different dimensions including vascularity, pigmentation, acceptability in patients’ perspective, comfort in observers’ perspective and contour. An overall score can be obtained by summing up scores from different items. A higher score suggests a more severe scar. Assessment results from this scale is highly associated with observers and thus shall be seen as moderately reliable.
3. Patient and Observer Scar Assessment Scale (POSAS) [26]
  POSAS consists of two numerical scales: The Observer Scar Assessment Scale and the Patient Scar Assessment Scale (Fig. 1). The Observer Scar Assessment Scale assesses six variables: vascularity, pigmentation, thickness, relief, pliability, and surface area. The Patient Scar Assessment Scale assesses six variables: pain, itching, color, stiffness, thickness, and relief. The major advantage of the POSAS is the inclusion of patient’s self-assessment.

**Table 1:** The Vancouver Scar Scale

Variables	Contents	Scores
Pigmentation	Normal—color that closely resembles the color over the rest of one’s body	0
	Hypopigmentation	1
	Mixed pigmentation	2
	Hyperpigmentation	3
Thickness	Normal	0
	< 1 mm	1
	≥ 1 mm and ≤ 3mm	2
	>3 mm and ≤ 4 mm	3
	> 4 mm	4
Vascularity	Normal—color that closely resembles the color over the rest of one’s body	0
	Pink	1
	Red	2
	Purple	3
Pliability	Normal	0
	Supple (flexible with minimal resistance)	1
	Yielding (giving way to pressure)	2
	Firm (solid, inflexible, not easily moved, resistant to manual pressure)	3
	Banding (rope-like tissue that blanches with extension of scar)	4
	Contracture (permanent shortening of scar producing deformity or distortion)	5

Despite continuous emerging and application of new scar assessment tools around the world, the above three scales are still the most widely accepted tools.

It should be noted that panel members reach an agreement that the parameters currently used in the scar scales are mainly subjective, resulting in limited reliability in short-term assessment and poor consistence in long-term evaluation. With advances in imaging technology, a variety of imaging devices with high-precision and high-resolution are used to provide a more objective evaluation of parameters such as color, texture, and thickness, thus enabling a more accurate scar assessment. Regretfully, devices available now for measurement still have major limits and more improvements are expected. Therefore, we still recommend a routine use of the international assessment scales, but the proportion of objective variables may increase appropriately. Hospitals equipped with advanced devices may realize objective measurements and assessments by leveraging imaging technologies.

## Prevention and treatment of scars

### Principles of scar prevention and treatment

#### Early intervention

The precise mechanism of excessive scarring is still unknown, and there is no ideal approach to cure scars after maturation. Hence, early interventions on scars are of great significance. Early intervention, defined as treatments and controls at the beginning of scarring after re-epithelialization, consists of two phases of management: prior-scarring phase and scarring phase. Early intervention is intended to reduce the risk of scar deterioration, namely, to inhibit scar growth via eliminating various factors that contribute to scarring. Many studies support that silicone-based products, pressure therapy, and topical agents (e.g., onion extract and some traditional Chinese medicine), when used alone or in combination, are effective approaches for early intervention with good tolerance and proven improvements in appearance and symptoms [27–29].

#### Combination therapy

Efficacy of monotherapy remains unsatisfactory due to the complexity of scarring mechanism and persistently evolving process. Clinical experience and available evidence-based data suggest that a combination approach using multiple modalities of different mechanisms and different categories (e.g., the combination of silicon-based products and onion extract-containing preparations, or agents and surgical interventions, or agents and laser therapy) is more effective [30–32]. But the optimal combination therapy is still to be investigated and determined. With the development of clinical technologies, novel therapeutic approaches will emerge, thus enabling the improvement of strategies on scar management.

#### Persistent therapy

Scarring is a long and progressive process, requiring persistent and adequate treatments. Regular assessment is a key factor in the whole treatment process, which includes assessment on the scar growth and the efficacy of prior treatments. A persistent and dynamic treatment algorithm should be developed based on the assessments until treatment satisfaction is achieved.

### Scar prevention

Scar prevention should be initiated immediately after skin injury occurs to reduce the risk of excessive scarring, the importance of which is not less than treatment. It shall be necessary to assess the risk of scarring and to take presentational efforts based on risk stratification.

#### Risk assessment on scarring

Large-scale study defining the risk stratification of scarring is still lacking. Cautious consideration of the risk factors contributing to scarring in the clinical evaluation is an alternative approach to help determine risk stratification of scarring. Female, young age [33], deep dermal injury, injury extending through all layers of the skin, large total body surface area (TBSA) from burn or trauma [34], tension site [33], long healing time (more than 3 weeks) [3], acid burn [35], repeated ulceration, infection and multiple surgical procedures, meshed skin grafts [33], postoperative infection, previous inappropriate treatments, and other latrogenic factors are recognized risk factors for scarring, coming either from summary of clinical experiences or from proven clinical study results.

Members of the panel reach a consensus that individuals who meet any of the conditions as follows should be considered as high-risk population: having history of hypertrophic or keloid scarring, undergoing procedure on high-risk sites (e.g., breast, thorax), having a family history of pathologic scarring, or having more than one risk factors (excludes gender and age) mentioned above. Individuals who meet all conditions as follows should be considered as low-risk population: no history of hypertrophic or keloid scarring, no history of procedures on high-risk sites (e.g., breast, thorax), no family history of pathologic scarring, or without any of the risk factors (excludes gender and age) mentioned above. Individuals between these two situations mentioned above should be considered as moderate-risk population.

#### Therapeutic options for scar prevention

Scar prevention includes prevention before scarring initiation and prevention before scar maturation. Prevention before scarring initiation should focus on wound care and surgical procedures. Preventing and controlling infections, creating favorable conditions for wound healing, and closing the wound as soon as possible are key points in would care optimization. Preventive options related to surgical procedures include sterile principle, non-(minimally) invasive technique, no tension, no foreign matter, no dead cavity, appropriate timing, and type of the procedure. Taking actions before scar maturation may still inhibit scar growth to a certain extent, reduce severity of scarring, and mitigate impairments of the body. Therapeutic options are pressure therapy [9, 36], drugs [9, 37, 38], radiotherapy [39], photodynamic therapy [40], and comprehensive function rehabilitation therapy. Patients should be stratified through risk assessment before treatment selection. Specific recommendations are summarized as follows:

*High-risk patients*

- *Recommendation 1*: Silicone-based products and pressure therapy should be used in combination as soon as possible after wound healing (epithelialization) and up to scar maturation.
- *Recommendation 2*: Silicone gel in cream or ointment form may be preferable to silicone gel sheeting for high-mobility or large areas, the face, or in humid climates.
- *Recommendation 3*: Adopting agents in ointment form including onion extract-containing preparations and some Chinese medicine may achieve better compliance than silicone gel sheeting or pressure therapy.
- *Recommendation 4*: For small scars getting unsatisfactory efficacy from prior treatments and rapidly worsened, concurrent intralesional corticosteroid injections are warranted.
- *Recommendation 5*: For widespread burn scars, regular use of photodynamic therapy in concurrent is recommended.
- *Recommendation 6*: For highly vascularized scars, the concurrent use of photodynamic therapy is recommended in addition to the above therapies.

*Moderate-risk patients*

- *Recommendation 1*: Silicone-based products, onion extract-containing preparations, pressure therapy and Chinese medicines for external application should be used alone or in combination.
- *Recommendation 2*: Agents that are more likely for patients to adhere should be selected based on individualized consideration of injury location, income and educational level.

*Low-risk patients*

- *Recommendation 1*: Standard hygiene practices are advised.
- *Recommendation 2*: Silicone-based products, onion extract-containing preparations, and some Chinese medicines for external application may be applied if patients have concerns on scarring.

*Additional considerations for scar prevention*

There is little evidence regarding the effect of solar ultraviolet irradiation on the cosmetic appearance of scar tissue. However, one study showed that postoperative sun exposure aggravates the clinical appearance of cicatrices [41]. Sunscreen was proposed as primary method to protect skin from direct sunlight in a clinically relevant animal model [42]. Scars should not be exposed to sunlight during the healing period, and sun protection is advised.

*Prevention of scar carcinoma*: Scar carcinoma can be induced by repeated scar ulcer [43]. In severe cases, skin biopsy is recommended as soon as possible for persistent stubborn and chronic scarring wounds, to determine pathological features and thus laying a basis for subsequent treatment. Surgical excision is recommended as soon as possible in these cases. Skin graft and flap should be used to achieve complete wound repair.

### Scar treatment

Treatment selection are determined by scar classification, patient history of scar (including previous treatment successes or failures), as well as the compliance to therapies. The presence of symptoms, most frequently pain or pruritus, may necessitate a specific treatment course or adjuvant therapy.

Therapies and agents now available include topical preparations (onion-extract [44–46], mitomycin C [47], imiquimod [48]), intralesional injections (bleomycin [6], corticosteroids [8], 5-fluorouracil (5-FU) [49]), physical therapy (silicone-based products [50], radiotherapy [39], cryotherapy [51], pressure therapy [52] and hypoallergenic microporous tape [53]), surgery and photodynamic therapy (intensive pulsed light, pulsed dye laser, fractional laser, and radiofrequency ablation) [54].

#### Hypertrophic scar treatment

Strategies for managing hypertrophic scars are based on scar types including immature or erythematous hypertrophic scars, linear hypertrophic scars arising from surgery or trauma, and widespread burn hypertrophic scars. Specific recommendations are summarized as follows:

*Immature or erythematous hypertrophic scars*

- *Recommendation 1*: Use of silicone gel, hypoallergenic paper tape, and onion extract–containing formulations is advised as preventive approach.
- *Recommendation 2*: In the case of persistent erythema (for more than 1 month) despite prevention efforts, treatment should be converted to that of a linear hypertrophic scar (see linear hypertrophic scars arising from surgery or trauma for details), or alternatively, laser therapy including pulsed dye laser therapy and fractional laser therapy may be applied.

*Linear hypertrophic scars arising from surgery or trauma*

- *Recommendation 1*: Silicone-based products, pulsed dye laser therapy, or fractional laser therapy is the preferred therapy in proliferation phase. Fractional laser therapy can also be applied in maturation phase, noting that better outcomes can be achieved via ablative fractional laser than non-ablative fractional laser.
- *Recommendation 2*: Adjunctive intralesional injection of corticosteroid or 5-FU is indicated when continuous use of silicone gel or sheeting is proved to be non-effective or unsatisfactory, and/or when severe scar hyperplasia and pruritic are observed.
- *Recommendation 3*: Pressure therapy may also be applied when scars are not resolved with agents mentioned in recommendation 1. Pressure therapy alone is unlikely to be sufficient.
- *Recommendation 4*: If a longer treatment duration (e.g., 12 month) of conservative therapy is unsuccessful, surgical excision can be applied. Appropriate actions should be taken to prevent recurrence based on the postoperative risk stratification of scars.
- *Recommendation 5*: When contraction is significant and scarring creates functional impairment, surgical excision to relieve tension should be considered. Z-plasty or W-plasty is appropriate to reduce scar tension and reduce the risk of recurrence. A wave incision or S-plasty can also be used in linear hypertrophic scar reconstruction with favorable results.
- *Recommendation 6*: Skin graft or local flap may be used in the treatment of larger linear hypertrophic scars. Adjuvant therapy is advised after surgery to prevent scar recurrence, but no single therapy has emerged as the primary treatment option.
- *Recommendation 7*: For severe scars, two treatment options are available. First option is surgical excision combined with injection of triamcinolone in different layers, and subsequent monthly corticosteroid administration. Another option is monthly intralesional injection of 5-FU and corticosteroid, or treatment with new agents like bleomycin or mitomycin C.

*Hypertrophic burn scars*

**Fig. 2:**
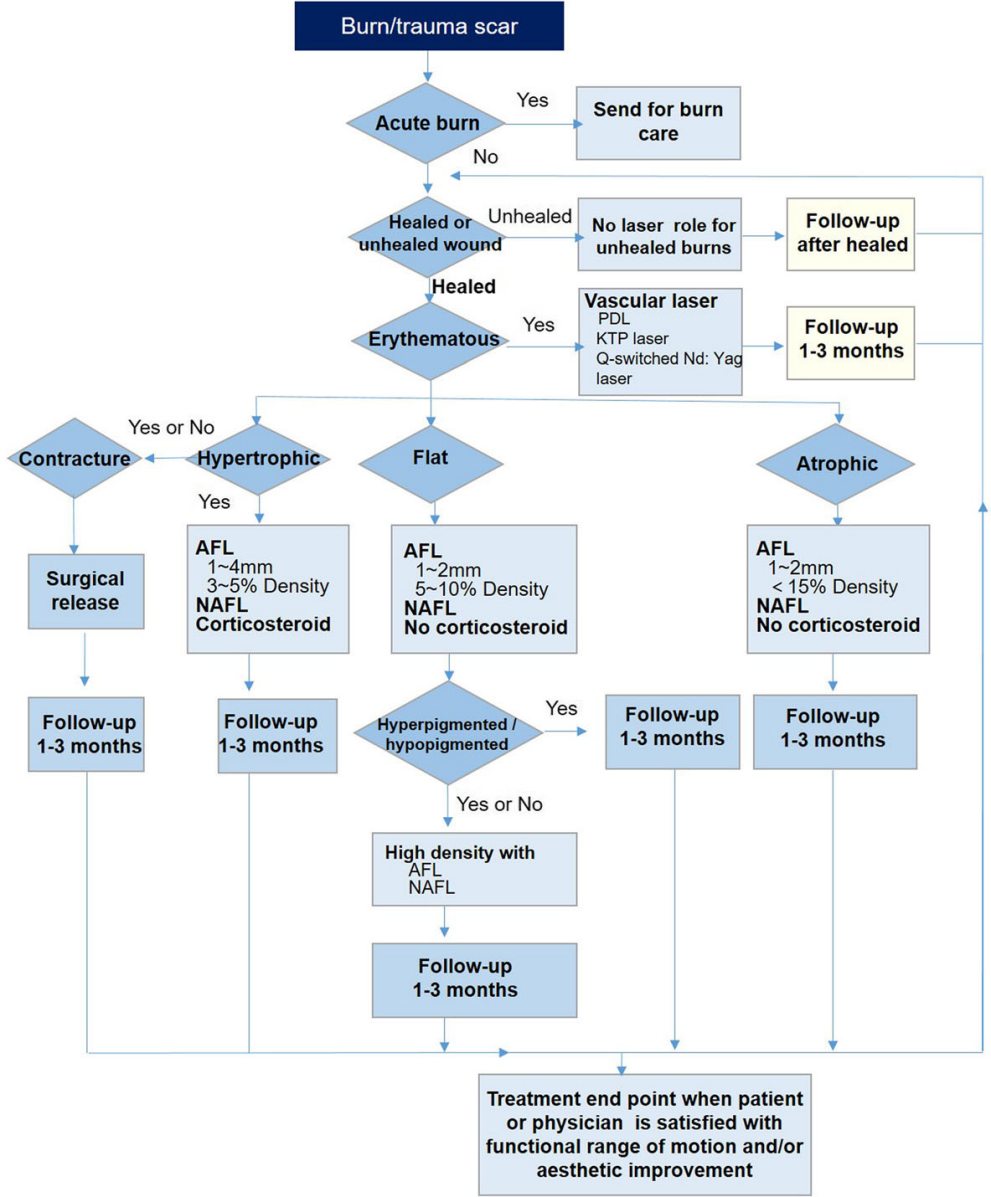
Laser treatment algorithm recommended for burn/trauma scar. *AFL* ablative fractionated laser, *KTP* potassium titanyl phosphate, *NAFL* nonablative fractionated laser, *PDL* pulsed-dye laser

- *Recommendation 1*: Patients with widespread burns should be admitted to a special burn unit for treatment and care. Once the epithelium is intact and stable, scar prevention and treatment should be initiated.
- *Recommendation 2*: Use of silicone gel preparations is the preferred fist-line therapy and can be used in combination with pressure therapy or onion-extract containing formulations.
- *Recommendation 3*: More emphasis should be put on the appropriate application of laser therapy as early prevention and treatment for burn and traumatic scars. (A) When erythema occurs in patients with burn and traumatic scars, laser therapy should be initiated as soon as possible. Yet, proper management after cautious evaluation on healing, contracture, and acute ulcer is a necessity. (B) Fractional laser therapy (including ablative and non-ablative) and vascular laser therapy (pulsed dye laser therapy, neodymium-yttrium-aluminum-garnet laser therapy, potassium-titanyl phosphate laser therapy and intensive-pulsed-light devices) are options for alternate or combined use. (C) Ablative fractional laser therapy requires fewer sessions than non-ablative fractional laser therapy. (D) The laser treatment algorithm for burn and traumatic scars is illustrated as Fig. 2.
- *Recommendation 4*: The clinical algorithm for prevention and treatment of burn scars is complex and often require individualized treatment consisting of combination or alternative therapies including silicone gel sheeting, individualized pressure therapy, massage/physical therapy or both, corticosteroid application, laser therapy, and surgical procedures. Massage, hydrocolloids, and antihistamines may be added to the therapeutic regimen to relieve pruritus.

*Keloids treatment*

The consensus panel believes that Chinese patients with keloids usually have a tendency to more serious scar physique, more aggressive scarring process, more severe, and more susceptible to recurrence, when compared to Caucasians. Under this circumstance, the treatment strategy proposed by our peer is based on Chinese clinical practice and has proven clinical efficacy. Key principles are as follows:

- *Recommendation 1*: A treatment regimen should consider patients’ age and distinguish between adult and pediatric patients. The following recommendations are applicable for adults, while children should refer to guideline specialized for pediatric patients.
- *Recommendation 2*: Surgical excision with subsequent prevention is the preferred approach. Anti-tension therapy, radiotherapy, and chemotherapeutic agents may largely control the keloid relapse after surgery. For larger keloids that cannot be closed directly after surgery, adjunctive solutions with skin flap, soft tissue expanders, and skin graft should be considered for wound healing.
- *Recommendation 3*: Non-surgical therapy can be considered as the preferred therapy for minor keloids and “inflammatory type” keloids. Intralesional injection of mixed formula that contains corticosteroids and other drugs, in combination with other concurrent therapeutic options, is recommended to avoid recurrence.
- *Recommendation 4*: Chemotherapeutic agents should be a key component in keloid injection regimen to avoid recurrence. Intralesional 5-FU is recommended as a primary choice.
- *Recommendation 5*: Radiotherapy after surgical excision is a first-line therapy to prevent recurrence of keloid in adults.
- *Recommendation 6*: Conservative physical therapy is the preferred option for pediatric patients.

*Atrophic scar treatment*

The panel believes that treatment strategy for atrophic scars should be based upon lesion site, initial injury or primary disease. Generally, photodynamic therapy, intralesional injections, surgical excision, and topical preparations are current therapeutic options; however, evidence available is not enough to support a priority for any of the mentioned options [55]. For atrophic scars after acne infection, which has high treatment demands, the number of treatment evidence and expert experience is relatively rich. Specific recommendations are summarized as follows:

- *Recommendation 1*: Treatment for atrophic acne scars generally requires a combination of multiple therapeutic options to achieve satisfactory results.
- *Recommendation 2*: Laser therapy is first-line therapy for atrophic acne scars, and fractional laser therapy achieves better outcomes.
- *Recommendation 3*: In atrophic acne scars with concavity as main features, tissue augmentation can be applied.
- *Recommendation 4*: Chemical peeling and surgical excision will require selection of a complex treatment strategy, such as type of the surgical procedures, refining of the procedures, selection of the chemical peels, etc. An individualized treatment strategy based on patient’s baseline characteristics and surgeon’s personal experience is recommended.

*Scar carcinoma treatment*

Surgery is the most effective approach, including amputations and extended focal resection [43, 56]. Presence of distal metastasis should be investigated before the surgery. Wound repair after surgical excision should be personalized regarding the location, area and depth of carcinoma, patient’s condition, surgeon’s experience, etc.

## Conclusion

Scar still remains a difficult issue on a world-wide scale. The assessment and treatment of scars is a continuous process. An accurate assessment on different individuals at different stages is the basis for the determination of appropriate treatment strategies, whereas developing effective, convenient, and repeatable scar assessment scales may precede further investigations in this area. With deepening of the researches on scars, the emergence of novel treatment concepts and techniques brings hope to the scar management, thus enabling improvements to scar prevention and treatment guidelines. Since a consensus report is a document for academic guidance and clinical evidence is an important basis, therapeutic strategies based on extensive evidences are the main recommendations of this report. Chinese medicine, regarded as the treasure of our traditional culture, has a long history in clinical use and abundant empirical evidences. More clinical trials are expected to investigate the efficacy and safety of Chinese medicines in the near future.

## Abbreviations

5-FU: 5-fluorouracil
AFL: Ablative fractionated laser
KTP: Potassium titanyl phosphate
NAFL: Nonablative fractionated laser
PDL: Pulsed-dye laser
POSAS: Patient and Observer Scar Assessment Scale
TBSA: Total body surface area
VAS: Visual Analog Scale
VSS: Vancouver Scar Scale
